# Addressing desaturation in a tracheal stenosis patient using the transnasal humidified rapid-insufflation ventilatory exchange technique during tracheostomy: A case report

**DOI:** 10.1097/MD.0000000000034567

**Published:** 2023-08-04

**Authors:** Sou Hyun Lee, Eunyoung Cho, Ji Hoon Park, Jae Yun Lee, Ji Hee Hong, Hyeji Han

**Affiliations:** a Department of Anesthesiology and Pain Medicine, Keimyung University School of Medicine, Daegu, Republic of Korea.

**Keywords:** airway obstruction, anesthesia, case reports, continuous positive airway pressure, tracheostomy

## Abstract

Transnasal humidified rapid-insufflation ventilatory exchange (THRIVE) is used in tracheostomy but not in cases of airway obstruction. This case report explores the use of THRIVE for managing airway obstruction during tracheostomy in patients with subglottic and tracheal stenosis, thereby addressing the current knowledge gap and exploring its potential for airway management.

**Patient concerns:**

A 63-year-old female with subglottic and tracheal stenoses underwent tracheostomy. Multiple attempts to establish a patent airway were unsuccessful, and oxygen saturation dropped to 56%.

**Diagnosis:**

Endotracheal tube was directed toward the tracheal wall, causing airway obstruction.

**Interventions:**

THRIVE was administered to the patient. Subsequently, the tube position was adjusted to enhance ventilation.

**Outcomes:**

The patient’s oxygen saturation increased to 99%. The postoperative complications, including subcutaneous emphysema, pneumothorax, pneumomediastinum and pneumopericardium, resolved. The patient was discharged on postoperative day 9.

**Lessons:**

THRIVE could be considered a temporary measure to enhance oxygenation before initiating a definitive treatment strategy.

## 1. Introduction

In 2014, transnasal humidified rapid-insufflation ventilatory exchange (THRIVE) through a high-flow nasal cannula (HFNC) was developed and introduced as a technique for apneic oxygenation to extend the apnea time between anesthesia induction and airway establishment.^[1]^ Ensuring airway patency is crucial for effective oxygen delivery,^[2]^ and although previous studies have implemented THRIVE in patients with upper airway lesions during tracheostomy, the procedures were performed while the patients were either conscious or under sedation to ensure airway patency.^[3,4]^ To date, the use of THRIVE to manage airway obstruction during tracheostomy has not been investigated, representing a gap in current knowledge and practice. In this case report, we present a novel approach using THRIVE to manage a patient who experienced desaturation due to an obstructed airway while undergoing tracheostomy to treat subglottic and tracheal stenoses. This case report highlights the potential of the THRIVE system as an effective tool for maintaining oxygenation in challenging situations, thereby expanding its application in the field of airway management.

## 2. Case report

This manuscript adhered to the Case Report (CARE) guidelines.

**Figure 1.**
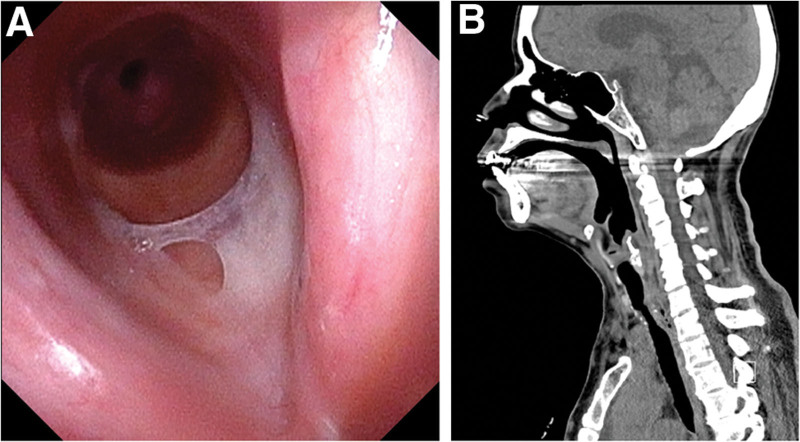
(A) Preoperative fiberoptic laryngoscopy image showing Mayer–Cotton grade 3 subglottic stenosis. (B) Preoperative neck computed tomography (sagittal view) showing tracheal stenosis.

A 63-year-old female (weight: 45.5 kg, height: 155.6 cm, body mass index: 18.8 kg/m^2^) with a medical history of diabetic end-stage renal disease and trachea stenosis due to intubation for 2 weeks following a cardiac arrest entered the operation room for tracheostomy under general anesthesia. Preoperative echocardiography revealed a severe decrease in left ventricular function (left ventricular ejection fraction < 40%), while fiberoptic laryngoscopy (Fig. 1A) and neck computed tomography (Fig. 1B) indicated the presence of Mayer–Cotton grade 3 subglottic stenosis with tracheal stenosis located 4.5 cm below the vocal cords, with the narrowest diameter measuring 6.0 mm. However, prior to surgery, the patient’s noninvasive blood pressure and peripheral oxygen saturation levels were normal. On the day of surgery, the patient was monitored using a pulse oximeter, 3-lead electrocardiography, and a noninvasive blood pressure device.

Following preoxygenation with 100% oxygen and induction, a size 6.0 endotracheal tube was selected for intubation. Intubation was attempted using video laryngoscopy with a grade 1 Cormack–Lehane view. While the tip of the endotracheal tube passed smoothly through the vocal cords, it did not advance further. Consequently, a second attempt was made using a size 3 i-gel laryngeal mask airway (LMA). Upon LMA placement, pressure-controlled ventilation began with a pressure of 18 cmH_2_O, respiratory rate of 16 breaths/min, and tidal volumes ranging between 220 and 240 mL. After placing the drape, the surgeon performed surgical tracheostomy at a level above the tracheal stenosis. Initially, a Portex cuffed tracheostomy tube was used (length: 6.5 cm, size: 7.0); however, it failed to pass through the required distance. Although the ventilator circuit was reconnected to the LMA, no tidal volume was achieved. A 6.5-sized endotracheal tube was subsequently inserted at the tracheostomy site; still, mechanical ventilation did not result in tidal volume or end-tidal CO_2_ (ETCO_2_) measurements.

Despite the attending anesthesiologist switching to manual ventilation using a bag and achieving peak pressures of up to 50 cmH_2_O, the patient’s condition did not improve, and oxygen saturation levels declined to 56%. The endotracheal tube remained in place; however, the LMA was removed. Subsequently, THRIVE (Optiflow; Fisher & Paykel, Auckland, New Zealand) was administered transnasally at a rate of 60 L/min and fraction of inspired O_2_ of 1.0, with the mouth securely taped closed. This intervention led to an increase in oxygen saturation levels to 99%. A 22-G left dorsalis pedis artery catheter was inserted for continuous blood pressure monitoring, and the first arterial blood gas analysis (ABGA) was performed. Although 4 attempts were made using endotracheal tubes of various sizes (6.5, 5.5, 5.0, and 4.5) through the tracheostomy site, every attempt to manually ventilate the patient through the inserted tube resulted in elevated airway pressures (up to 50 cmH_2_O) and low tidal volumes (50–100 mL), with no detection of ETCO_2_; lung auscultation revealed no audible breathing sounds.

**Figure 2.**
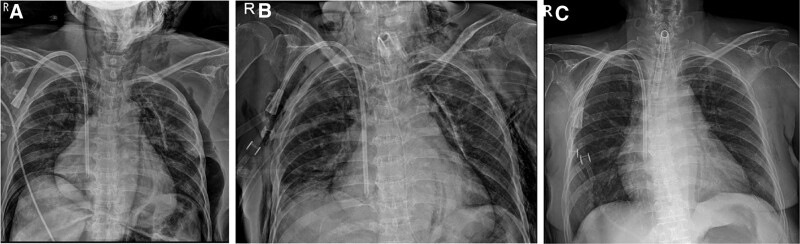
(A) Intraoperative chest X-ray. (B) Chest X-ray taken 1 day postoperatively. (C) Chest X-ray taken 3 days postoperatively.

A portable chest radiograph was obtained following the second ABGA, revealing that the endotracheal tube inserted through the tracheostomy site could not bypass the tracheal stenosis; the tube was instead directed toward the tracheal wall (Fig. 2A). Following a thorough evaluation, it was concluded that repositioning the Portex tube opening above the stenosis would be advantageous. Accordingly, the Portex tube was inserted superficially by the surgeon, securing it at a depth of 5 cm and ultimately leading to effective ventilation with the detection of ETCO_2_ (30 minutes duration after the onset of desaturation).

**Figure 3.**
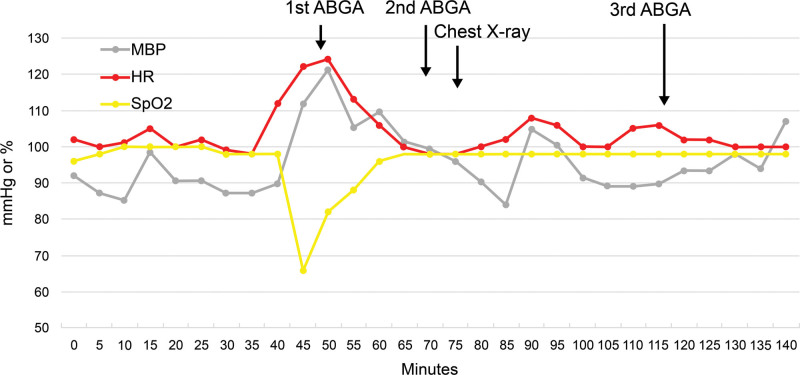
Trends in heart rate (HR), mean arterial pressure (MAP), and peripheral oxygen saturation (SpO_2_) during anesthesia.

The third ABGA test was performed, and the results after applying HFNC are described in Table 1. The patient’s blood pressure and heart rate remained stable throughout the surgery (Fig. 3), after which the patient was transferred to a general ward. Postoperative chest radiography revealed extensive subcutaneous emphysema, bilateral pneumothorax, pneumomediastinum, and pneumopericardium (Fig. 2B). A chest tube was inserted into the right chest, and follow-up chest radiography on postoperative day 3 showed that these issues had been resolved (Fig. 2C). The patient was discharged from the hospital with a retainer on postoperative day 9.

**Table 1:** Arterial blood gas analysis results.

	1st ABGA	2nd ABGA	3rd ABGA
pH (mEq/L)	7.071	6.965	7.246
PaCO_2_ (mm Hg)	102	Unmeasurable	61.3
PaO_2_ (mm Hg)	211	131	98.4
HCO_3_^–^ (mmol/L)	29.7	Unmeasurable	26.6
BE (mmol/L)	−2.9	Unmeasurable	−1.6
SatO_2_ (%)	88.2	96.1	96.7
Hemoglobin (g/dL)	12.6	12.2	11.5
Hematocrit (%)	38.5	37.3	35.3
Lactic acid (mmol/L)	0.3	0.2	0.3

ABGA = arterial blood gas analysis, BE = base excess, PaCO_2_ = partial pressure of carbon dioxide, PaO_2_ = partial pressure of oxygen, SatO_2_ = oxygen saturation.

## 3. Discussion

The THRIVE method involves the delivery of high-flow (60–70 L/min) 100% oxygen that is humidified and heated to 37°C, administered through nasal cannulae. Although THRIVE has proven useful in providing apneic oxygenation to patients with upper airway obstructions resulting from general anesthesia, it is crucial to ensure that the airway remains unobstructed during airway surgical procedures, such as tracheostomy.^[5,6]^

The main findings of this case report demonstrate the potential effectiveness of THRIVE for managing a patient with an obstructed airway during tracheostomy for subglottic and tracheal stenosis. THRIVE led to increased oxygen saturation, and the patient successfully tolerated the procedure without any desaturation incidents.

In our comparison with previous research, we found parallels with studies regarding the efficacy of THRIVE in patients with subglottic stenosis who underwent balloon dilation under general anesthesia.^[7–9]^ While these reports also indicated no desaturation events during periods of 13–22 minutes, the patients in those studies did not experience airway obstruction during the procedure, and THRIVE was utilized to prevent, rather than treat, desaturation, as observed in the current case. Impressively, even when THRIVE was applied after the initial desaturation event in this situation, the patient was successfully sustained for 30 minutes without any additional desaturation incidents. In the current case, THRIVE was used in a situation involving an obstructed airway; still, oxygen saturation increased to 99% with a PaO_2_ of 211 mm Hg. Since airway pressure can reach 7 cmH_2_O when O_2_ is administered at 40 L/min with the mouth closed,^[10]^ increasing the flow to 60 L/min with the mouth closed can lead to a higher airway pressure. Consequently, this allowed oxygen to be conveyed through the gap between the endotracheal tube and tracheal wall.

A key strength of our report was the successful use of THRIVE in a challenging situation involving an obstructed airway. A body mass index <25 kg/m^2^ also allowed the patient to better tolerate potential desaturation.^[11]^ Additionally, this case highlights the potential role of THRIVE as a temporary measure for enhancing oxygenation before initiating a definitive treatment strategy. This finding contrasts with that of a previous report stating that once a patient experiences desaturation, THRIVE cannot be considered a reliable method for increasing oxygen saturation.^[3]^ A key limitation in our case was the unmeasurable extent of PaCO_2_ levels, which prevented us from confirming the increase in the PaCO_2_ rate and making comparisons with other studies (with PaCO_2_ rates of 2.0 mm Hg/min).^[12]^ This limitation is closely related to the obstructed airways. Nevertheless, no adverse events, such as brain injury or cardiac failure, occurred, even when the PaCO_2_ level exceeded 100 mm Hg.^[13]^

In this case report, we demonstrated the potential effectiveness of THRIVE in managing a patient with an obstructed airway during tracheostomy for subglottic and tracheal stenosis. Although our findings highlighted the role of THRIVE as a temporary measure to enhance oxygenation before initiating a definitive treatment strategy, its long-term effectiveness in these situations remains unclear. It is crucial to acknowledge that the observed conditions, including subcutaneous emphysema, pneumothorax, pneumomediastinum, and pneumopericardium, are more likely to be attributed to increased ventilatory pressure^[14,15]^ due to a poorly positioned tracheostomy tube or damage to the tracheal wall during the procedure, rather than the use of HFNC. This conclusion is supported by the fact that these conditions typically occur in patients with compromised pulmonary interstitial tissues, resulting in alveolar air leaks.^[16,17]^ Furthermore, as described by Zhou et al,^[18]^ venovenous extracorporeal circulation may be the sole life-saving procedure in cases of airway obstruction and respiratory failure. This alternative procedure should be considered in the management of patients with airway obstruction, as our case report suggests that THRIVE can be employed as a temporary measure to improve oxygenation; however, its long-term effectiveness in such situations remains to be investigated.

In conclusion, this case report highlights the potential of THRIVE as a temporary measure to enhance oxygenation in patients with airway obstruction during tracheostomy. However, venovenous extracorporeal circulation should be considered as a life-saving alternative in cases involving airway obstruction and respiratory failure, as although THRIVE may provide temporary oxygenation improvement, its long-term effectiveness requires further investigation.

## Acknowledgment

We would like to thank Editage (www.editage.co.kr) for English language editing.

## Author contributions

**Conceptualization:** Sou Hyun Lee.

**Data curation:** Hyeji Han.

**Writing – original draft:** Sou Hyun Lee, Eunyoung Cho.

**Writing – review & editing:** Ji Hoon Park, Jae Yun Lee, Ji Hee Hong.
